# Immobilization of Naringinase onto Polydopamine-Coated Magnetic Iron Oxide Nanoparticles for Juice Debittering Applications

**DOI:** 10.3390/polym16233279

**Published:** 2024-11-25

**Authors:** Scott D. Kimmins, Antonella Henríquez, Celia Torres, Lorena Wilson, Marcos Flores, Edgar Pio, Domingo Jullian, Bruno Urbano, Stephanie Braun-Galleani, Carminna Ottone, Lisa Muñoz, Martha Claros, Paulina Urrutia

**Affiliations:** 1 Instituto de Química, Pontificia Universidad Católica de Valparaíso, Valparaíso 2373223, Chile; scott.kimmins@pucv.cl (S.D.K.); antonella.henriquez.a@mail.pucv.cl (A.H.); lisa.munoz@pucv.cl (L.M.); 2 Escuela de Ingeniería Bioquímica, Pontificia Universidad Católica de Valparaíso, Valparaíso 2340000, Chile; torresmartinezcelia@gmail.com (C.T.); lorena.wilson@pucv.cl (L.W.); stephanie.braun@pucv.cl (S.B.-G.); carminna.ottone@pucv.cl (C.O.); 3 Laboratory of Surface and Nanomaterials, Physics Department, Faculty of Mathematical and Physical Sciences, University of Chile, Santiago 8330111, Chile; mflorescarra@uchile.cl; 4 Instituto de Ciencias de la Ingeniería, Universidad de O’Higgins, Rancagua 2841959, Chile; edgar.pio@uoh.cl (E.P.); domingo.jullian@uoh.cl (D.J.); 5 Departamento de Polímeros, Facultad de Ciencias Químicas, Universidad de Concepción, Concepción 3349001, Chile; burbano@udec.cl; 6 Departamento de Ingeniería Metalúrgica y de Materiales, Universidad Técnica Federico Santa María, Valparaíso 2390123, Chile; martha.claros@usm.cl

**Keywords:** enzyme immobilization, naringinase, polydopamine, magnetic nanoparticles, juice debittering

## Abstract

Chemical amination of the enzyme was demonstrated to favor immobilization onto polydopamine (PDA)-coated magnetic nanoparticles (MNPs) for the first time, to the best of the author’s knowledge. MNPs prepared via hydrothermal synthesis were coated with PDA for the immobilization of naringinase. X-ray diffraction, transmission electron microscopy, X-ray photoelectron spectroscopy, and Fourier-transform infrared spectroscopy showed that the MNPs were composed mainly of Fe_3_O_4_ with an average size of 38.9 nm, and coated with a 15.1 nm PDA layer. Although the specific activities of α-L-rhamnosidase (RAM) and β-D-glucosidase (GLU) of free naringinase decreased with amination, the immobilization yields of the aminated enzyme increased by more than 40% for RAM and more than 10-fold for GLU. The immobilization improved the enzyme’s thermal stability (at 50 °C), reaching a half-life of 40.7 and 23.1 h for RAM and GLU activities, respectively. The biocatalyst was successfully used for the debittering of grapefruit juice, detecting a reduction in naringin of 56% after 24 h. These results demonstrate that the enzyme amination is an effective strategy to enhance the immobilization on a PDA coating and could be applied to other enzymes in order to obtain an easily recoverable biocatalyst using a simple immobilization methodology.

## 1. Introduction

**Figure 1:**
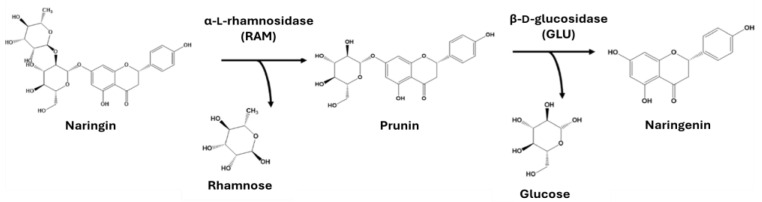
Hydrolysis of naringin by α-L-rhamnosidase (RAM) and β-D-glucosidase (GLU) activities of naringinase.

The control of citrus juice bitterness is critical to product quality and value. Naringin, a flavanone glycoside, is a major bitter compound in grapefruit, bitter orange, and pomelo [1]. Various techniques are used to debitter citrus juice, including physico-chemical and biochemical methods. The enzymatic hydrolysis of naringin via naringinase is notable for its selectivity and ability to retain the juice’s nutritional properties [2,3]. Naringinase catalyzes the hydrolysis of naringin to naringenin in a two-step reaction [2]. Firstly, the α-L-rhamnosidase (EC 3.2.1.40; RAM) activity takes place by producing prunin (4,5,7-trihydroxy flavanone-7-glucoside) and rhamnose. Secondly, the β-D-glucosidase (EC 3.2.1.21; GLU) activity is responsible for hydrolyzing prunin to naringenin (4,5,7-trihydroxy flavanone) and glucose (Figure 1).

Naringinases of various origins have been used in juice debittering, mainly fungal [4,5,6], but also from bacteria of terrestrial [7] and marine [8] sources. Among the naringinases from fungi, different *Aspergillus* strains have been studied, including the enzyme from *Aspergillus aculeatus* and *Aspergillus niger*. The *A. aculeatus* naringinase is a multimeric enzyme of 348 kDa, containing four subunits (100, 95, 84, and 69 kDa), where the three larger subunits are GLU and only the smallest subunit is an RAM. Its optimal pH is 4.0 and the optimal temperature is 50 °C [4]. Naringinase from *A. niger* is also a multimeric enzyme, but it has two subunits of around 65.5 kDa, with an optimal pH of 4.4–5.0 and optimal temperature range of from 45 to 55 °C [9].

Immobilized enzymes have several advantages that make them attractive to the food processing industry. The biocatalyst can be reused, making its operation conditions more flexible and enabling the development of continuous processes. In addition, the catalyst can be stabilized, thus extending its lifetime and resulting in an enzyme-free product [10,11,12]. However, immobilization can also have some drawbacks, such as loss of enzyme activity, additional costs due to the immobilization process, unwanted changes in the kinetic properties of the enzyme, and limitations in mass transfer [13,14]. These limitations can be addressed by developing new or improved immobilization strategies [15,16,17,18,19]. In this context, naranginase has been immobilized on several materials using different methodologies. The use of natural polymeric materials, such as agarose [19,20], alginate [21,22], and chitosan [23,24], has the advantage of producing large particle sizes in the mm range, which facilitates the biocatalyst recovery step. The main disadvantage of these particles is their low mechanical stability, which increases the risk of product contamination. Recently, the use of inorganic supports with good mechanical properties, such as zeolites [25] and mesoporous silica [26], has been studied. Nevertheless, the separation of the biocatalyst from the reaction medium remains a significant hurdle due to the small size of the supports (at the nanometric or micrometric scale). To overcome this challenge, the use of magnetic particles has emerged as an alternative for the recovery of the biocatalyst.

The predominant iron oxides found in nature are magnetite (Fe_3_O_4_), maghemite (γ-Fe_2_O_3_), and hematite (α-Fe_2_O_3_) [27,28]. When reduced to the nanoscale, these ferromagnetic materials exhibit superparamagnetism, a form of magnetism that occurs only under the influence of an external magnetic field [29], enhancing their practical utility due to the ease of separation. This kind of nanostructure has been studied in a plethora of applications, i.e., energy storage [30], bioimaging [31], sensors [32,33], water treatment [34], and targeted drug delivery [29,35].

The synthesis of iron oxide magnetic nanoparticles (MNPs) has been carried out using various methodologies, including electrodeposition [36], thermal decomposition [37,38,39], sol–gel processes [40,41], chemical precipitation [39], and hydrothermal methods [27,29,42]. Among these, the hydrothermal method is especially promising due to its relative simplicity and ability to produce highly crystalline nanopowders in a single step without the need for surfactants. Additionally, it allows for precise control over the nanoparticles’ size, shape, and crystallinity, making it an ideal method for producing high-quality Fe_3_O_4_ and γ-Fe_2_O_3_ nanoparticles [42,43]. 

MNPs are attractive supports for enzyme immobilization due to their high surface area, large surface-to-volume ratio, and easy recovery. Their use also leads to the challenges of high reactivity and susceptibility to degradation when exposed to certain environments [44,45]. However, several methods have been developed to modify MNPs in order to improve the properties of the support and prevent degradation, with the added advantage that the functionalization of MNPs provides the versatility required to tailor the MNPs for specific applications [46,47,48]. Several hybrid supports with an inorganic core covered by a polymeric bed have been proposed for enzyme immobilization [49,50]. The use of polymers in enzyme immobilization has been reported as an effective strategy for the stabilization of multimeric enzymes due to their interaction with the different subunits [51,52]. In the case of naringinase, it was immobilized on MNPs modified with polyethyleneimine, using glutaraldehyde as a crosslinking agent [53]. Even though good results were obtained in terms of activity and reusability, the toxicity of glutaraldehyde [54] is a limitation when the biocatalyst is intended for food production applications [55]. An alternative agent that allows for the covalent immobilization of enzymes, which does not exhibit cytotoxic effects, is polydopamine (PDA) [56,57].

A universal mussel-inspired PDA coating for a wide range of surfaces was first demonstrated over 17 years ago [58]. The coating was created by simply immersing the material in a basic aqueous solution of dopamine.HCl. The benefit of this methodology, apart from its simplicity and universality, is that it forms a relatively stable and reproducible nanometer-thick coating. Importantly, the coating remains chemically reactive and can be functionalized with a range of amine- and thiol-containing biomolecules [59,60,61], via Schiff base or Michael additions. PDA-coated surfaces have been used to covalently immobilize enzymes [59]. Naringinase has also been immobilized on a PDA/polyethyleneimine (PEI)-coated mesoporous material using glutaraldehyde as crosslinking agent and showed high activity with good stability/reusability. Over 75% of enzymatic activity was retained after 30 days storage, compared to only 30% for free naringinase, and, in addition, a relative activity of 60.8% was observed after eight cycles for the immobilized naringinase. Even though these results demonstrate the utility of the PDA immobilization method [62], as mentioned above, the use of glutaraldehyde as a crosslinking agent is a disadvantage when the biocatalyst is intended for use in food production due to its toxicity [54]. 

The presence of amino and thiol groups on the enzyme’s surface is a crucial factor in the immobilization of the enzyme on PDA-coated surfaces. Chemical amination represents an alternative way of modifying an enzyme’s surface, providing new anchoring points for the enzyme to conjugate to support. This type of amination has been carried out by activating superficial carboxylic groups (terminal carboxylic, Asp, and Glu) with 1-ethyl-3-(dimethylamino-propyl) carbodiimide (EDAC) in the presence of ethylenediamine (EDA) [63,64]. This strategy has already been employed to improve the activity and stability of enzymes immobilized on supports functionalized with aldehyde groups, and recently was shown to be a good alternative to improve the immobilization of naringinase in glyoxyl–agarose [19]. To the best of our knowledge, this strategy has not been reported previously for enzymes immobilized onto supports functionalized with PDA.

The development of a robust biocatalyst in terms of its expressed activity and thermal stability is crucial for its application at an industrial scale. The immobilization of naringinase is a complex process, as it must ensure the expression and stabilization of the RAM and GLU activities of the biocatalyst, and subsequently the conversion of naringin to naringenin. In this work, we show a simple and effective immobilization of naringinase on an iron oxide MNP surface functionalized with PDA without using any crosslinking agent. Chemical amination of the enzyme was used as a strategy to improve the biocatalyst’s expressed activities, and the benefits of the polydopamine coating and MNPs (*vide supra*) are further applied for the debittering of citrus juices in the food industry.

## 2. Materials and Methods

### 2.1. Materials

A naringinase complex from *Aspergillus aculeatus* and *Aspergillus niger* (Novozyme NS 33117) was kindly provided by Novozyme (Copenhagen, Denmark). The following reagents were provided by Sigma-Aldrich (Darmstadt, Germany): dopamine hydrochloride, Tris base, p-nitrophenyl-α-L-rhamnopyranoside (pNPR), p-nitrophenyl-β-D-glucoside (pNPG), 1-ethyl-3-(3-dimethylaminopropyl) carbodiimide (EDAC), and ethylenediamine (EDA). Ferrous chloride tetrahydrate (FeCl_2_∙4H_2_O, purity > 99%) was purchased from LobaChemie (Mumbai, India). Ferric chloride hexahydrate (FeCl_3_∙6H_2_O, purity > 99%) and ammonium hydroxide (NH_4_OH, 25% ammonia) were purchased from Winkler Ltd.a (Santiago, Chile). All the reagents were used without purification and other chemicals were of analytical grade. Grapefruits were acquired in the local market and handmade grapefruit juice was used for debittering tests.

### 2.2. Analytical Methods

#### 2.2.1. Determination of Naringinase Complex Activities 

The naringinase complex was characterized in terms of RAM and GLU activities. One international unit of RAM (IU_RAM_) or GLU (IU_GLU_) was defined as the amount of enzyme that produces 1 µmol of pNP per minute from 5 or 10 mM of pNPR or pNPG solution, respectively, at pH 4.0 and 45 °C. Typically, 100 µL of the catalyst solution or suspension was added to 0.9 mL sodium citrate buffer 25 mM containing 5 mM pNPR or 10 mM pNPG (45 °C; pH 4.0). Samples of 100 µL were collected every 5 min and added to 3 mL of NaOH (0.5 M) to stop the reaction. Finally, the absorbance was measured at 405 nm (6715 UV/Vis Jenway Spectrophotometer). The extinction coefficient of pNP was 32.815 M^−1^ cm^−1^. The protein concentration was determined by the Bradford method, using bovine serum albumin as a standard.

#### 2.2.2. Quantification of Bitter Compounds Concentration

The concentration of bitter compounds was assessed by DAD-HPLC analysis (JASCO-DAD HPLC) according to Muñoz et al. [20]. Naringin was detected at 280 nm and prunin and naringenin at 210 nm. A C-18 analytical column (15 cm × 0.4 cm) was used for separation at 35 °C and the injection volume was 20 µL. The flow rate of the mobile phase was 0.5 mL min-1 using a gradient of acetonitrile in water of 5:95 (*v*/*v*) for 4 min, 5:95 to 40:60 (*v*/*v*) in 10 min, 40:60 (*v*/*v*) for 2 min, 40:60 to 70:30 (*v*/*v*) in 8 min, 70:30 to 5:95 (*v*/*v*) in 4 min, and 5:95 (*v*/*v*) for 4 min.

#### 2.2.3. X-Ray Photoelectron Spectroscopy (XPS)

XPS spectra were collected using a PHI1250 photoelectron spectrometer (Physical Electronics, Chanhassen, MN, USA) with an Al Kα X-ray source (hν = 1486.71 eV). To minimize aging and contamination effects, samples were stored in a nitrogen atmosphere, beginning no more than an hour after preparation. The analysis chamber operated in a vacuum at less than 1 × 10^−6^ mbar while measurements were made. X-ray photoelectron spectroscopy data were analyzed with PHI Multipak software (V9.4.0.7). The energy scale of the spectra was calibrated relative to the binding energy (BE) of adventitious hydrocarbons (C-C/C-H) in the C1s signal at 284.8 eV. Curve fitting and decomposition were performed after Shirley-type background removal. A mixed Gaussian–Lorentzian line shape was used for the different components.

#### 2.2.4. X-Ray Diffraction (XRD)

The XRD patterns of the MNPs were recorded on a Bruker D8 Advance (Karlsruhe, Germany) in Bragg–Brentano configuration with Co-Kα radiation (λ = 1.7889 Å, coupled to a 0.1 mm Fe filter, 35 kV, 35 mA) using an Eiger 2R 500 K detector in 1D mode. The XRD patterns were obtained in 2θ ranges between 30° and 80° with a step size of 0.02° and holding time of 0.7 s/step. Phase identification was carried out using the DIFFRAC.EVA.7 software (V7) from BRUKER, with the PDF-2 2004 database. Quantitative analysis was performed using the Materials Analysis Using Diffraction (MAUD) software (V.2.1) [65,66], where LaB6 (a = 4.1565 (1) Å) was used as an external standard for determining instrumental broadening [67]. Two phases were indexed: Fe_3_O_4_ (PDF 89-2355) and Fe_2_O_3_-γ (PDF 39-1346).

#### 2.2.5. Transmission Electron Microscopy (TEM) 

The size distribution of MNPs was determined utilizing TEM (JEM 1200 II, JEOL, Tokyo, Japan). MNPs were dispersed in a dissolution of 1:1 ethanol–water and sonicated for 5 min. Then, a dispersion droplet was placed in a copper grid and the solvent was evaporated before the analysis. The size and distribution of unmodified MNPs were obtained by determining the diameter of at least 300 nanoparticles using the software ImageJ (version 1.54g). The size and distribution of PDA-modified MNPs was obtained by determining the thickness of the coating of at least 100 nanoparticles using the software Image J (version 1.54j). 

#### 2.2.6. Fourier Transform Infrared Spectroscopy (FT-IR)

The FT-IR spectra were acquired with a Jasco V4600 instrument (Tokyo, Japan) equipped with an ATR module. The samples were dried at 60 °C overnight, and the analyses were performed at room temperature (range 4000–500 cm^−1^, 64 scans, resolution 2 cm^−1^).

### 2.3. Chemical Amination of Naringinase Complex 

Chemical amination of the enzyme was performed according to the previously reported methodology [68]. First, 5 mL of the enzymatic solution (11 mg L^−1^ of protein) was added to 45 mL of EDA solution 1 M at pH 4.75 and 5 °C. EDAC was added to a concentration of 9 mM and mixed for 30 min. The solution was then dialyzed at 5 °C in a 10 mM sodium phosphate buffer pH 7.0. 

### 2.4. Support Synthesis and Functionalization

#### 2.4.1. Magnetic Nanoparticle Synthesis

MNPs were synthesized through hydrothermal synthesis following the procedure previously described [43], with slight changes. An equimolar quantity of 37.5 mmol of FeCl_2_∙4H_2_O and FeCl_3_∙6H_2_O was dissolved in 45 mL of distilled water and 25 mL of ammonium hydroxide were added while maintaining constant stirring for 5 min. The solution was poured into a Teflon-lined autoclave vessel (100 mL) and maintained at 150 °C for 24 h. Afterwards, the autoclave vessel was allowed to cool naturally to ambient temperature and the product was separated using an external magnetic field and washed first with water and then with ethanol. Finally, the product was dried at 90 °C for 5 h. 

#### 2.4.2. Polydopamine Functionalization of Magnetic Nanoparticles 

A total of 2.50 g of MNPs was weighed using a watch glass and added to a round-bottom flask containing 500 mL of 10 mM Tris buffer solution at pH 8.5. The MNPs were then sonicated for 15 min to ensure that no precipitates remained on the glass of the round-bottom flask. Subsequently, 1.25 g of dopamine hydrochloride was added under stirring and vigorously mixed for 3 h using an overhead stirrer; the pH was maintained at 8.5 with the addition of 1 M HCl. The buffer solution was removed via magnetic decantation and the MNPs were then washed five times with 250 mL of ultrapure water, sonicated for 15 min, and finally washed again with 250 mL of ultrapure water, with each wash lasting 30 min. The modified MNPs were then stored in 100 mL of ultrapure water at 4 °C until further use.

### 2.5. Enzyme Immobilization

Unmodified and chemically aminated catalysts were immobilized by adding 1 g of PDA-MNP to 10 mL of enzyme solution (1 mg protein mL^−1^) at 20 °C for 24 h. Enzyme solutions were prepared in a phosphate buffer of 25 mM, pH 7. At the end of the immobilization, the catalyst was separated from the medium via an external magnetic field and washed three times with the buffer used to measure activity. A suspension of the catalyst in the activity buffer was then prepared and its RAM- and GLU-specific activities were measured and expressed as IU_RAM_/g and IU_GLU_/g.

The immobilization process was defined by the following parameters [69]: immobilization yield in terms of activity (IY_RAM_; IY_GLU_) and protein (IY_P_) and expressed activity (a_RAM_; a_GLU_). These are presented in Equations (1)–(3).

(1) $${IY}_{RAM}=\frac{a_{RAM}}{{RAM}_{0}}\cdot 100$$

(2) $${IY}_{GLU}=\frac{a_{GLU}}{{GLU}_{0}}\cdot 100$$

(3) $${IY}_{P}=\frac{P_{I}}{P_{0}}\cdot 100$$

where P_I_ is the immobilized protein (difference in protein concentration of the supernatant at the start and end of the immobilization process), P_0_ is the offered protein (protein at the start of the immobilization process), a_RAM_ and a_GLU_ are the RAM- and GLU-specific activities of the immobilized biocatalyst (assessed by suspending the immobilized biocatalyst in the activity buffer), and RAM_0_ and GLU_0_ are the RAM and GLU activities offered per gram of support, respectively. Student’s *t*-tests were conducted to evaluate differences in immobilization parameters.

### 2.6. Influence of Temperature on Initial Reaction Rate

A total of 100 µL of the catalyst solution or suspension was added to 0.9 mL sodium citrate buffer 25 mM containing 5 mM pNPR or 10 mM pNPG pH 4.0 at different temperatures (30–75 °C). Samples of 50 µL were collected every 5 min over a 30 min period and were added to 3 mL of a 0.5 M NaOH solution to stop the reaction. The absorbance was determined at 405 nm (6715 UV/Vis, Jenway Spectrophotometer, Staffordshire, United Kingdom). The initial reaction rate was expressed as µmole of pNP per minute. 

### 2.7. Thermal Stability of Biocatalysts

The thermal stabilities of the soluble and immobilized catalysts were evaluated at 50 °C in 25 mM sodium citrate buffer pH 4.0. Periodically, solution or suspension samples were collected to evaluate RAM and GLU activities. Residual activities are expressed as the ratio between the activity at different inactivation times and the initial activity of the biocatalyst. Inactivation kinetics were modeled according to a first-order mechanism with residual activity, according to Equations (4) and (5) [70]:

(4) $$E\;k\to \;E_{1}$$

(5) $$e/e_{0}=(1-\alpha )\cdot exp(-k\cdot t)+\alpha$$

where *E* is the active enzyme state, *E*_1_ is the final state of the enzyme with non-zero specific activity, *k* is the first-order rate constant of enzyme inactivation, *e*/*e*_0_ represents the residual activity at time *t* (quotient of the enzyme activity at time *t* and *t*_0_), and *α* is the ratio of specific activities between the final and initial enzyme species [70]. Parameters of the inactivation model were determined via nonlinear regression using Microsoft Excel Solver (V. 2410). Half-life (*t*_1/2_), which is defined as the time at which the enzyme has lost one-half of its initial activity, is calculated according to Equation (6):

(6) $$t_{1/2}=ln\left(\frac{1-\alpha }{0.5-\alpha }\right)\cdot \frac{1}{k}$$

Stability factors in terms of RAM and GLU activities were defined as the ratio of the *t*_1/2_ of immobilized and soluble biocatalysts at 50 °C and pH 4.0.

### 2.8. Grapefruit Juice Debittering

The debittering of grapefruit juice was carried out by offering 2 IU_RAM_ per mL of juice. The juice was centrifuged prior to the experiment, and the reaction was carried out in a light-covered 25 mL flask at 150 rpm and 50 °C. Periodically, 200 µL samples of the reaction medium were collected and centrifuged and the supernatant was filtered (0.22 µm pore size) for analysis via HPLC.

The reusability of the biocatalyst was assessed via the grapefruit juice debittering experiments, conducted in repeated batches and lasting 24 h each. After each batch, the catalyst was separated from the juice via the application of an external magnetic field and washed with distilled water. The catalyst was then reused in a fresh reaction batch, maintaining the initial catalyst-to-juice mass ratio. The stability of the immobilized catalyst under reactive conditions was evaluated by measuring the RAM and GLU catalysts’ residual activities after three reaction batches.

## 3. Results and Discussion

### 3.1. Support Synthesis and Functionalization

**Figure 2:**
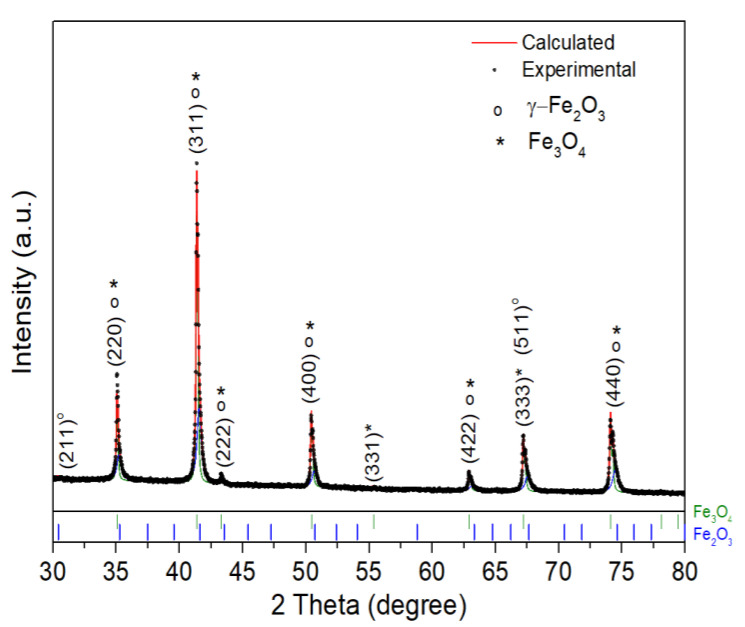
Rietveld refinement of the X-ray diffraction patterns of the magnetic nanoparticles. The experimental data (dots) and modeling results (colored line), as well as the difference between experimental and calculated patterns, are presented below. The peaks corresponding to γ-Fe_2_O_3_ and Fe_3_O_4_ are identified with the symbols ο and *, respectively.

Figure 2 presents the results obtained from the XRD analysis of MNPs, where the Rietveld refinements of the XRD patterns determined a composition of 54% of Fe_3_O_4_ and 46% of γ-Fe_2_O_3_ (in wt.%). The Delf line broadening model and an isotropic size–strain model in MAUD were used to model the microstructure of the phases [71,72]. The resulting spectra have characteristic peaks corresponding to the cubic spinel structure of magnetite (PDF# 82-1533). However, the presence of the maghemite phase is also noticeable (PDF# 39-1346). Differentiating both phases using XRD is usually challenging since both have the same cubic structure and their lattice parameters are almost identical [73], which can also be seen in these results.

Therefore, it is proposed that during the hydrothermal process, the reactions that may occur are the formation of the magnetite (Fe_3_O_4_), followed by in situ oxidation leading to maghemite (γ-Fe_2_O_3_), which can be formulated according to Equations (7) and (8) [74,75]:

Fe^2+^ + 2Fe^3+^ + 8OH^−^ → Fe_3_O_4_ + 4H_2_O (7)

4Fe_3_O_4_ + O_2_ → 6(γ − Fe_2_O_3_) (8)

**Figure 3:**
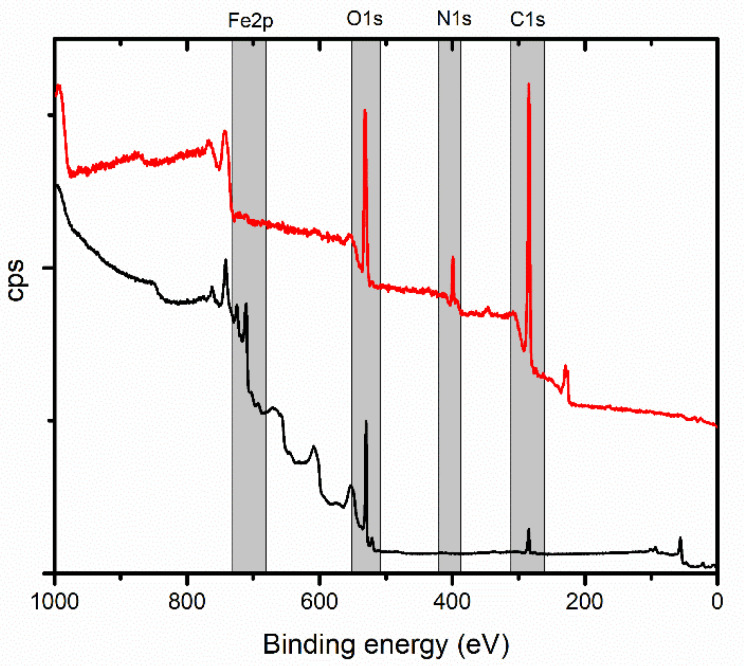
Low-resolution X-ray photoelectron spectroscopy (XPS) spectra of the magnetic nanoparticle samples (black), and polydopamine-coated magnetic nanoparticles (red).

The XPS spectra of the MNPs (black line) and PDA-MNPs (red line) are shown in Figure 3. In addition to the iron, oxygen, and carbon peaks, the nitrogen peak is also visible for the PDA MNP sample. The amount of carbon contamination is not negligible, as shown by the intensity of the C 1s peak in the spectrum of the MNPs sample. Most of the carbon is adventitious carbon (aliphatic carbon), which was acquired during sample preparation and handling. The MNPs mainly constitute Fe^2+^ and Fe^3+^ (707.9 and 710.9 eV, respectively), and the XPS spectrum of PDA-MNPs exhibited prominent peaks corresponding to C 1s (284.5 eV) and N 1s (399.4 eV). The fitting of the C 1s and O 1s signals can be interpreted/attributed to the presence of the PDA molecules. In the case of the N 1s signal, a single peak is observed at a binding energy of 399.4 eV, related to the N-C bond. From the XPS spectrum, the calculated nitrogen-to-carbon (N/C) ratio of the PDA-MNPs was determined to be 0.102, which is close to the theoretical N/C ratio of 0.125 for dopamine [76]. The composition of the PDA-MNPs closely aligns with the expected theoretical values, indicating the successful coating and incorporation of PDA onto the MNPs.

**Figure 4:**
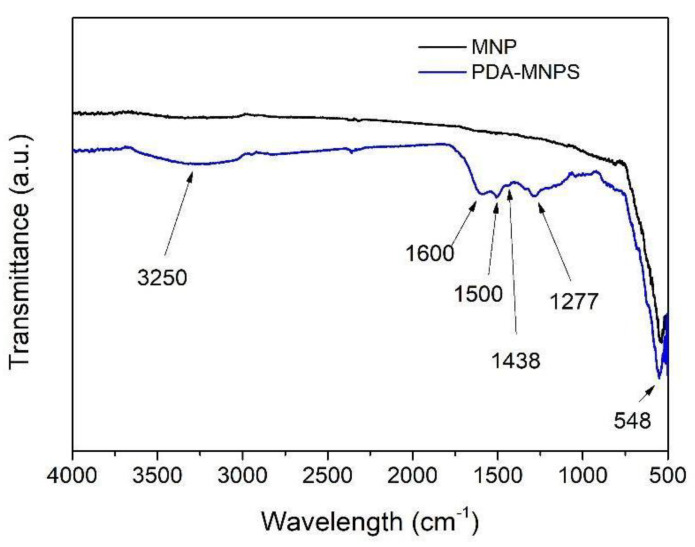
FT−IR spectra of magnetic nanoparticles (MNPs) and polydopamine-coated magnetic nanoparticles (PDA-MNPs).

The successful coating of the MNPs was also confirmed by FT-IR (Figure 4). The MNPs show a smooth spectrum with only the M-O-M characteristic band of Fe_3_O_4_ materials at 548 cm^−1^ [77]. The PDA coating on the MNP surface can be confirmed via the appearance of the typical bands observed for dopamine [78]. The transmittance band in the range 3000–3600 cm^−1^ is associated with the aromatic O-H stretching vibration and the peak at 1600 cm^−1^ is attributed to the aromatic C=C stretching vibration of the phenol structure. The wide peak at 1600 cm^−1^, together with the 1500 cm^−1^, 1438 cm^−1^, and 1277 cm^−1^ peaks, are attributable to the -NH_2_ deformation vibrations. The other bands observed between 1500 and 600 cm^−1^ are typically observed for PDA and are attributable to CH_2_ bending vibrations, C-O-H asymmetric bending vibrations, C-O asymmetric vibrations, and C-N stretching vibrations. 

**Figure 5:**
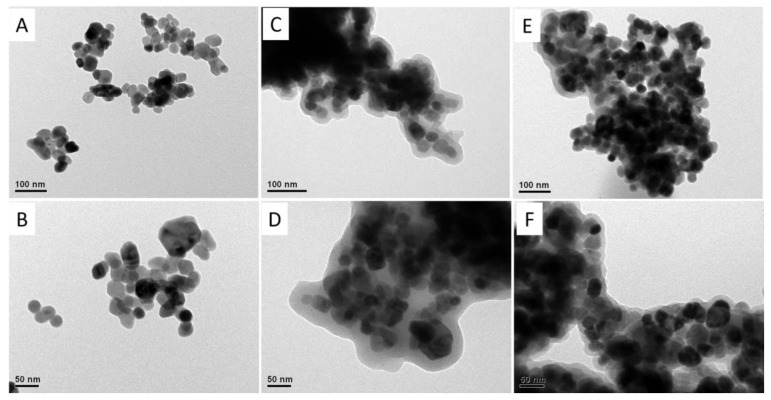
TEM images of (**A**,**B**) magnetic nanoparticles, (**C**,**D**) polydopamine-coated magnetic nanoparticles, and (**E**,**F**) aminated naringinase immobilized on PDA-MNPs. Scale bars (**A**,**C**,**E**) = 100 nm; scale bars (**B**,**D**,**F**) = 50 nm.

The size and morphology of the iron oxide MNPs, PDA-MNPs, and aminated naringinase immobilized onto PDA-MNPs (Naringinase-PDA-MNPs) were observed via TEM (Figure 5). Unmodified MNPs were composed of nanoparticle clusters of 38.9 ± 14.9 nm in diameter. It is shown that the PDA-MNPs are aggregated and coated with a PDA layer of 15.1 ± 5.5 nm in thickness (Figure 5C,D). Finally, TEM images of Naringinase-PDA-MNPs (Figure 5E,F) showed a small increase in aggregation with respect to the PDA-coated MNPs. This has been observed before in the literature with lipase-immobilized PDA MNPs and it is not clear if this occurs during the immobilization step or if it originates from the TEM sample preparation [76].

**Figure 6:**
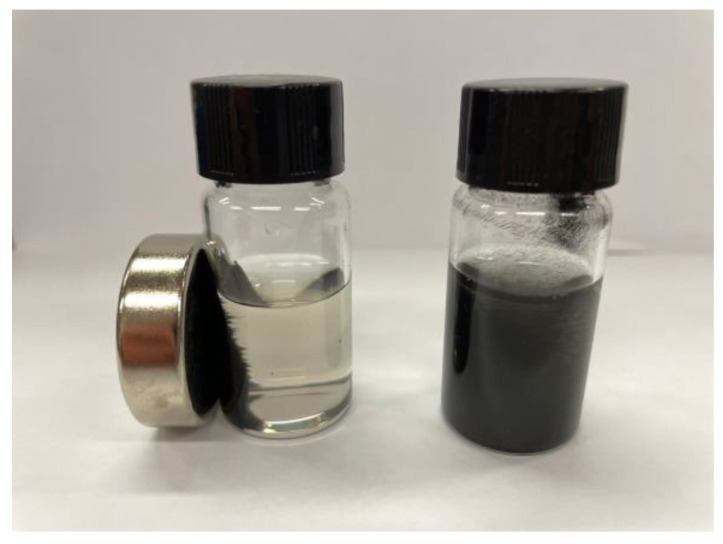
Photograph of an aqueous suspension of PDA-MNPs before (**right**) and after isolation with a magnet (**left**).

MNPs were chosen as a support for naringinase so that the biocatalyst could be easily recovered. Therefore, it was important to evaluate whether the magnetic properties were maintained after the deposition of PDA. As can be seen in Figure 6, the PDA-MNPs can be easily separated from an aqueous solution through the application of an external magnetic field.

### 3.2. Enzyme Immobilization

The naringinase complex was chemically aminated in order to increase the number of amino groups on the surface of the enzyme to enhance its reactivity with PDA. The chemical amination resulted in a loss of activities (Table 1), which may be related to the partial modification of Asp and Glu residues involved in the RAM and GLU catalytic mechanisms [79] and/or changes in the physical properties of the enzyme’s surface.

The unmodified and aminated enzymes were immobilized in PDA-MNPs and Table 2 displays the immobilization parameters. The catalyst obtained by the immobilization of the aminated enzyme showed a 2.3-fold improvement in IY_P_ compared to the non-aminated enzyme, showing that the amination step has a positive effect on the attachment of the enzyme complex to the PDA-MNPs. When IY was evaluated in terms of RAM and GLU activities, a significant difference between the unmodified catalyst and its aminated counterpart was obtained (*p* < 0.05), with the latter providing the best results. The IY was increased by more than 40% for RAM and more than 10-fold for GLU with the amination step. Moreover, the RAM and GLU activities expressed by the aminated immobilized catalyst were higher than the values obtained with the unmodified enzyme, despite the loss of activity caused by the chemical amination process. The positive effect of the chemical amination on naringinase immobilization has been reported previously when the enzyme was immobilized on glyoxyl-agarose [19], but was not previously evaluated for immobilization on PDA-coated carriers. 

### 3.3. Characterization of Immobilized Catalyst

The aminated enzyme immobilized onto PDA-MNPs was compared with its soluble counterpart in terms of the effect of temperature on the initial reaction rates of pNPR and pNPG hydrolysis, the thermal stability, and the performance in grapefruit juice debittering.

#### 3.3.1. Influence of Temperature on Initial Reaction Rate

**Figure 7:**
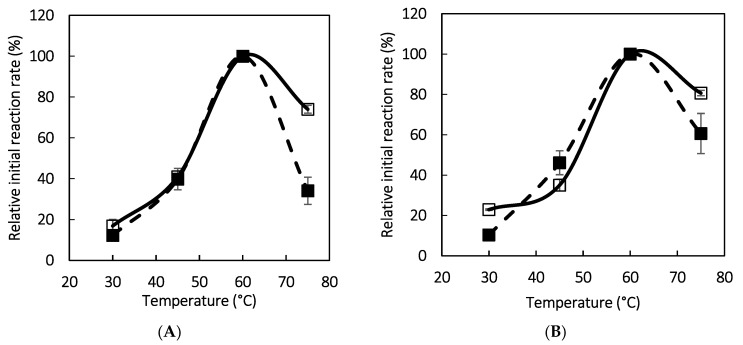
Effect of temperature on the initial reaction rate of pNPR (empty symbols) and pNPG (full symbols) hydrolysis at pH 4.0. (**A**) Soluble and (**B**) immobilized aminated naringinase.

Figure 7 shows the effect of temperature on the initial reaction rate of pNPR and pNPG hydrolysis. As can be observed, the optimal temperature for the soluble and immobilized biocatalysts is 60 °C, with no significant differences in the range of 30 to 60 °C. At 75 °C, the enzymes immobilized on PDA-MNPs showed a higher relative initial reaction rate than their soluble counterpart, especially in the case of pNPG hydrolysis, a result that may be associated with the higher thermal stability of the immobilized enzymes at this temperature. This change in temperature profile was previously observed for immobilized naringinase [19,20].

#### 3.3.2. Thermal Stability of Biocatalysts

**Figure 8:**
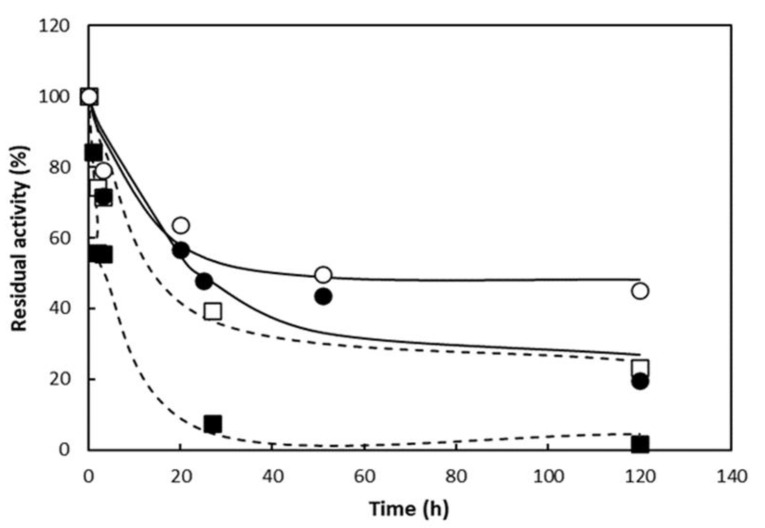
Thermal stability of soluble (square) and immobilized (circle) aminated naringinase at 50 °C and pH 4.0. α-L-rhamnosidase activity (empty symbol); β-D-glucosidase activity (full symbol).

In terms of the biocatalyst stability, the inactivation of the soluble and immobilized catalysts at 50 °C (Figure 8) showed a higher thermal stability in terms of RAM and GLU activities for the immobilized enzyme, results that may be associated with the increased rigidity of the enzyme structure due to the reaction with PDA-MNPs.

Inactivation kinetics were modeled according to a first-order inactivation mechanism with residual activity, and the parameters obtained are shown in Table 3.

In terms of RAM and GLU activities, the higher thermal stability is reflected in the lower inactivation rate constant and higher residual activity. After immobilization, the t_1/2_ of RAM and GLU increased, reaching a stability factor of 6.2 and 7.3, respectively. The stability factor obtained is of a similar magnitude to that reported by Bodakowska-Boczniewicz and Garncarek (2020), who achieved a t_1/2_ 11 times greater than that obtained with the soluble enzyme at 50 °C when using naringinase from *A. niger* crosslinked with dextran aldehyde [80]. The t_1/2_ values obtained with the catalyst immobilized on PDA-MNPs in this study are higher than the value reported by Ribeiro and Rabaça (2011) for naringinase from *Penicillium decumbens* immobilized as a crosslinked enzyme aggregate (4.5 h at 30 °C) [81].

#### 3.3.3. Grapefruit Juice Debittering

**Figure 9:**
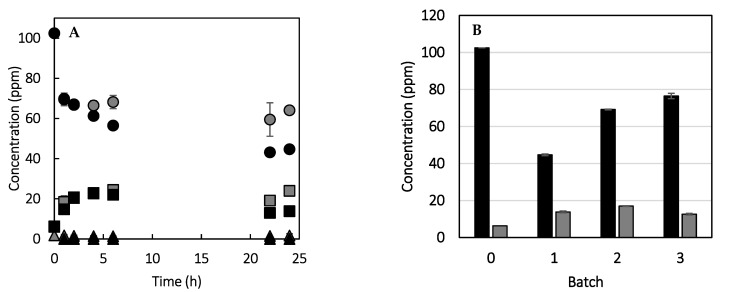
(**A**) Kinetics of grapefruit juice debittering catalyzed by soluble (gray) and immobilized (black) aminated naringinase: naringin (circle); prunin (square); naringenin (triangle). (**B**) Grapefruit juice debittering in repeated batch operations using aminated naringinase immobilized on polydopamine-coated magnetic nanoparticles. Concentration of bitter compounds after 24 h of reaction: naringin (black); prunin (gray). Reaction conditions: 2 IURAM per mL of juice; 50 °C; 150 rpm.

The performance of the soluble and immobilized catalysts in grapefruit juice debittering is shown in Figure 9A, obtaining a decrease in naringin concentration of 46% and 56% after 24 h of reaction, respectively. The decline in naringin concentration is more pronounced than that observed by Busto et al. when employing naringinase from *A. niger* immobilized on poly(vinyl alcohol) cryogels. In their investigation, the debittering efficacy of the simulated juice was evaluated at 20 °C, resulting in a 34% reduction in naringin after 24 h [82]. Other authors who immobilized naringinase from *Penicillium* species on alginate and chitosan observed different outcomes, achieving reductions in naringin concentration of 56 and 75%, respectively [6,83]. The kinetic of the first batch of reaction (Figure 9A) showed that as the concentration of naringin decreased throughout the reaction, the concentration of prunin increased, reflecting the effect of the catalysts’ RAM activity. The naringenin concentration obtained with the soluble enzyme after 24 h was 2 ppm, while no naringenin was detected in the case of the immobilized catalyst. The low conversion of prunin to naringenin was reported previously for the naringinase complex utilized [20] and may be related to the inhibition of the GLU activity due to the sugars present in the natural juice [83,84].

It can be observed that the maximum prunin concentration was obtained in the first 6 h of reaction, and in the case of the immobilized catalyst, its concentration decreased after 22–24 h of reaction. Given that the concentration of prunin achieved with the immobilized enzyme is less than the value that should be expected from a 56% decrease in naringin concentration, the enzyme-free support was evaluated for its ability to absorb naringin from the medium. This was achieved by incubating the enzyme-free support in grapefruit juice for a period of 24 h, maintaining a support–juice ratio consistent with that employed with the immobilized catalyst. It was found that the enzyme-free support reduced the initial naringin concentration by 39%, indicating that a portion of the naringin is adsorbed on the PDA-coated magnetic particles. This result could also explain the reduction in prunin concentration following a 22 h reaction period, since no naringenin was detected. 

The reutilization of the immobilized biocatalyst was evaluated through the analysis of three 24 h batches of grapefruit juice debittering (Figure 9B). It can be observed that the efficiency of the catalyst diminished with successive batches, which could be attributed to a reduction in the RAM activity due to its thermal inactivation, and also a decrease in the naringin that the support can adsorb due to its saturation. The RAM and GLU activities of the immobilized biocatalyst were measured after three reaction batches (72 h), obtaining residual values of 33.6 ± 2.9% and 13.4 ± 1.9%, respectively. The thermal stability of the biocatalyst was lower than that under non-reactive conditions, a result that may be associated with the lower pH of grapefruit juice (pH 3). The reaction temperature will influence the reusability of the biocatalyst, as reported previously [80]; consequently, it could be expected that a lower temperature and reaction time will improve the biocatalyst performance. It is noteworthy that although the immobilization of naringinase on a PDA-coated support has been previously reported [85], its efficacy in juice debittering has not been assessed. 

## 4. Conclusions

MNPs were successfully prepared via hydrothermal synthesis and coated with PDA for the immobilization of a naringinase complex. The support, before and after functionalization, was fully characterized and the chemical amination of the naringinase complex was evaluated as a strategy to improve the attachment of the enzymes to PDA-MNPs. The amination under the conditions evaluated favored the immobilization onto PDA-MNPs and significantly increased the immobilization yields and the specific activity of the immobilized catalyst for both RAM and GLU activities compared to the unmodified catalyst. These results demonstrate that the immobilization strategy used in this work is suitable for preserving the activity of enzymes with two different catalytic functionalities, as in the case of naringinase. The immobilization also improved the thermal stability of the enzyme, highlighting that the amination step could potentially be used for immobilizing other enzymes on PDA coatings. 

The biocatalyst’s ability to debitter grapefruit juice was assessed, with a reduction in naringin of more than 56% being achieved after 24 h. This reduction was attributed to two phenomena: enzymatic hydrolysis and physical adsorption to the support. According to the reaction kinetics, the biocatalyst’s RAM activity is reflected in its prunin production, with the maximum concentration being obtained after 6 h of reaction, unlike naringenin, which was not observed during the reaction. 

The developed immobilized naringinase biocatalyst has characteristics that are desirable for a catalyst with industrial applications: the PDA functionalization offers a straightforward immobilization methodology; the immobilized catalyst presents high specific activity and allows for the thermal stabilization of the soluble enzyme; and the catalyst can be easily recovered from the reaction medium using a magnetic field. All these characteristics make this immobilization strategy an interesting alternative to consider in citrus juice debittering.

## Figures and Tables

**Table 1:** Impact of amination conditions on α-L-rhamnosidase (RAM) and β-D-glucosidase (GLU) activities of the naringinase complex.

Free Catalysts	RAM Activity (mIU_RAM_ mg_prot_^−1^)	Residual Activity (%)	GLU Activity (mIU_GLU_ mg_prot_^−1^)	Residual Activity (%)
Unmodified	33.4 ± 2.7	100	756.2 ± 33.1	100
Aminated	10.8 ± 1.1	30	229.5 ± 22.1	28

**Table 2:** Characterization of the immobilization of unmodified and aminated catalysts on polydopamine-coated magnetic nanoparticles.

Catalysts	IY_P_ (%)	IY_RAM_ (%)	IY_GLU_ (%)	a_RAM_ (mIU_RAM_/g)	a_GLU_ (mIU_GLU_/g)
Unmodified	23.2 ± 4.5	19.3 ± 0.8	2.2 ± 0.1	35.5 ± 1.4	80.3 ± 4.3
Aminated	52.5 ± 1.7	28.2 ± 0.2	28.8 ± 0.8	41.2 ± 0.4	700.1 ± 19.5

IY_P_: immobilization yield in terms of protein; IY_RAM_: immobilization yield in terms of α-L-rhamnosidase (RAM) activity, IY_GLU_: immobilization yield in terms of β-D-glucosidase (GLU); a_RAM_: RAM activity expressed by the immobilized catalyst; activity; a_GLU_: GLU activity expressed by the immobilized catalyst.

**Table 3:** Thermal inactivation parameters of α-L-rhamnosidase (RAM) and β-D-glucosidase (GLU) activities of soluble and immobilized aminated naringinase immobilized on polydopamine-coated magnetic nanoparticles (PDA-MNPs) considering a first-order inactivation mechanism with residual activity at 50 °C.

Catalyst/Activity	k (h^−1^)	α	t_1/2_ (h)
Soluble/RAM	0.19	0.31	6.6
Soluble/GLU	0.22	0.00	3.2
Naringinase–PDA–MNPs/RAM	0.08	0.48	40.7
Naringinase–PDA–MNPs/GLU	0.05	0.27	23.0

k: first-order rate constant of enzyme inactivation, α: residual activity; t_1/2_: half-life.

## Data Availability

The original contributions presented in the study are included in the article, further inquiries can be directed to the corresponding author.

## References

[1] Csuti A., Sik B., Ajtony Z. (2024). Measurement of Naringin from Citrus Fruits by High-Performance Liquid Chromatography—A Review. Crit. Rev. Anal. Chem..

[2] Puri M., Marwaha S.S., Kothari R.M., Kennedy J.F. (1996). Biochemical Basis of Bitterness in Citrus Fruit Juices and Biotech Approaches for Debittering. Crit. Rev. Biotechnol..

[3] Puri M., Banerjee U.C. (2000). Production, Purification, and Characterization of the Debittering Enzyme Naringinase. Biotechnol. Adv..

[4] Chen Y., Ni H., Chen F., Cai H., Li L., Su W. (2013). Purification and Characterization of a Naringinase from Aspergillus Aculeatus JMUdb058. J. Agric. Food Chem..

[5] Bodakowska-Boczniewicz J., Garncarek Z. (2022). Naringinase Biosynthesis by Aspergillus Niger on an Optimized Medium Containing Red Grapefruit Albedo. Molecules.

[6] Bodakowska-Boczniewicz J., Garncarek Z. (2019). Immobilization of Naringinase from Penicillium Decumbens on Chitosan Microspheres for Debittering Grapefruit Juice. Molecules.

[7] Mukund P., Belur P.D., Saidutta M.B. (2014). Production of Naringinase from a New Soil Isolate, Bacillus Methylotrophicus: Isolation, Optimization and Scale-up Studies. Prep. Biochem. Biotechnol..

[8] Selim M.S., Abo Elsoud M.M., Sanad M.N.M.E., Elattal N.A., Rifaat H.M., Mohamed S.S. (2023). Enzymatic Debittering of Citrus Juices: Optimization, Modeling, and Characterization of Naringinase Production from Marine Bacillus Subtilis Strain BSnari. Biocatal. Agric. Biotechnol..

[9] Ni H., Chen F., Cai H., Xiao A., You Q., Lu Y. (2012). Characterization and Preparation of Aspergillus Niger Naringinase for Debittering Citrus Juice. J. Food Sci..

[10] Razzaghi M., Homaei A., Vianello F., Azad T., Sharma T., Nadda A.K., Stevanato R., Bilal M., Iqbal H.M.N. (2022). Industrial Applications of Immobilized Nano-Biocatalysts. Bioprocess Biosyst. Eng..

[11] Homaei A. (2015). Enzyme Immobilization and Its Application in the Food Industry. Advances in Food Biotechnology.

[12] Ottone C., Romero O., Aburto C., Illanes A., Wilson L. (2020). Biocatalysis in the Winemaking Industry: Challenges and Opportunities for Immobilized Enzymes. Compr. Rev. Food Sci. Food Saf..

[13] Bolivar J.M., Woodley J.M., Fernandez-Lafuente R. (2022). Is Enzyme Immobilization a Mature Discipline? Some Critical Considerations to Capitalize on the Benefits of Immobilization. Chem. Soc. Rev..

[14] DiCosimo R., McAuliffe J., Poulose A.J., Bohlmann G. (2013). Industrial Use of Immobilized Enzymes. Chem. Soc. Rev..

[15] Zhang Z., Du Y., Kuang G., Shen X., Jia X., Wang Z., Feng Y., Jia S., Liu F., Bilal M. (2022). Lipase-Ca^2+^ Hybrid Nanobiocatalysts through Interfacial Protein-Inorganic Self-Assembly in Deep-Eutectic Solvents (DES)/water Two-Phase System for Biodiesel Production. Renew. Energy.

[16] Cui J., Ren S., Lin T., Feng Y., Jia S. (2018). Shielding Effects of Fe^3+^-Tannic Acid Nanocoatings for Immobilized Enzyme on Magnetic Fe_3_O_4_@silica Core Shell Nanosphere. Chem. Eng. J..

[17] Du Y., Zhao L., Geng Z., Huo Z., Li H., Shen X., Peng X., Yan R., Cui J., Jia S. (2024). Construction of Catalase@hollow Silica Nanosphere: Catalase with Immobilized but Not Rigid State for Improving Catalytic Performances. Int. J. Biol. Macromol..

[18] Riaz R., Ashraf M., Hussain N., Baqar Z., Bilal M., Iqbal H.M.N. (2023). Redesigning Robust Biocatalysts by Engineering Enzyme Microenvironment and Enzyme Immobilization. Catal. Lett..

[19] Urrutia P., Arrieta R., Torres C., Guerrero C., Wilson L. (2024). Amination of Naringinase to Improve Citrus Juice Debittering Using a Catalyst Immobilized on Glyoxyl-Agarose. Food Chem..

[20] Muñoz M., Holtheuer J., Wilson L., Urrutia P. (2022). Grapefruit Debittering by Simultaneous Naringin Hydrolysis and Limonin Adsorption Using Naringinase Immobilized in Agarose Supports. Molecules.

[21] Tavernini L., Aburto C., Romero O., Illanes A., Wilson L. (2021). Encapsulation of Combi-CLEAs of Glycosidases in Alginate Beads and Polyvinyl Alcohol for Wine Aroma Enhancement. Catalysts.

[22] Wilson L., Illanes A., Romero O., Ottone C., Ferreira M.L. (2023). Chapter 17—Future Perspectives in Enzyme Immobilization. Biocatalyst Immobilization.

[23] Urrutia P., Bernal C., Wilson L., Illanes A. (2018). Use of Chitosan Heterofunctionality for Enzyme Immobilization: β-Galactosidase Immobilization for Galacto-Oligosaccharide Synthesis. Int. J. Biol. Macromol..

[24] Tavernini L., Ottone C., Illanes A., Wilson L. (2020). Entrapment of Enzyme Aggregates in Chitosan Beads for Aroma Release in White Wines. Int. J. Biol. Macromol..

[25] Carceller J.M., Galán J.P.M., Monti R., Bassan J.C., Filice M., Yu J., Climent M.J., Iborra S., Corma A. (2020). Covalent Immobilization of Naringinase over Two-Dimensional 2D Zeolites and Its Applications in a Continuous Process to Produce Citrus Flavonoids and for Debittering of Juices. ChemCatChem.

[26] Li Q., Zhang N., Sun X., Zhan H., Tian J., Fei X., Liu X., Chen G., Wang Y. (2021). Controllable Biotransformation of Naringin to Prunin by Naringinase Immobilized on Functionalized Silica. J. Chem. Technol. Biotechnol..

[27] Ali A., Zafar H., Zia M., ul Haq I., Phull A.R., Ali J.S., Hussain A. (2016). Synthesis, Characterization, Applications, and Challenges of Iron Oxide Nanoparticles. NSA.

[28] Wang F., Qin X.F., Meng Y.F., Guo Z.L., Yang L.X., Ming Y.F. (2013). Hydrothermal Synthesis and Characterization of α-Fe_2_O_3_ Nanoparticles. Mater. Sci. Semicond. Process..

[29] Samrot A.V., Sahithya C.S., Selvarani A.J., Purayil S.K., Ponnaiah P. (2021). A Review on Synthesis, Characterization and Potential Biological Applications of Superparamagnetic Iron Oxide Nanoparticles. Curr. Res. Green Sustain. Chem..

[30] Park J.W., Na W., Jang J. (2016). Hierarchical Core/shell Janus-Type α-Fe_2_O_3_/PEDOT Nanoparticles for High Performance Flexible Energy Storage Devices. J. Mater. Chem. A Mater. Energy Sustain..

[31] Teja A.S., Koh P.-Y. (2009). Synthesis, Properties, and Applications of Magnetic Iron Oxide Nanoparticles. Prog. Cryst. Growth Charact. Mater..

[32] Harraz F.A., Faisal M., Jalalah M., Almadiy A.A., Al-Sayari S.A., Al-Assiri M.S. (2020). Conducting Polythiophene/α-Fe_2_O_3_ Nanocomposite for Efficient Methanol Electrochemical Sensor. Appl. Surf. Sci..

[33] Touba S., Kimiagar S. (2019). Enhancement of Sensitivity and Selectivity of α-Fe_2_O_3_ Nanorod Gas Sensors by ZnO Nanoparticles Decoration. Mater. Sci. Semicond. Process..

[34] Abdullah N.H., Shameli K., Abdullah E.C., Abdullah L.C. (2019). Solid Matrices for Fabrication of Magnetic Iron Oxide Nanocomposites: Synthesis, Properties, and Application for the Adsorption of Heavy Metal Ions and Dyes. Compos. Part B.

[35] Defu Z., Ting Y., Jian Y., Shuang F., Shubiao Z. (2020). Targeting Strategies for Superparamagnetic Iron Oxide Nanoparticles in Cancer Therapy. Acta Biomater..

[36] Elrouby M., Abdel-Mawgoud A.M., El-Rahman R.A. (2017). Synthesis of Iron Oxides Nanoparticles with Very High Saturation Magnetization Form TEA-Fe(III) Complex via Electrochemical Deposition for Supercapacitor Applications. J. Mol. Struct..

[37] Cotin G., Kiefer C., Perton F., Ihiawakrim D., Blanco-Andujar C., Moldovan S., Lefevre C., Ersen O., Pichon B., Mertz D. (2018). Unravelling the Thermal Decomposition Parameters for The Synthesis of Anisotropic Iron Oxide Nanoparticles. Nanomaterials.

[38] Eom Y., Abbas M., Noh H., Kim C. (2016). Morphology-Controlled Synthesis of Highly Crystalline Fe_3_O_4_ and CoFe_2_O_4_ Nanoparticles Using a Facile Thermal Decomposition Method. RSC Adv..

[39] Guardia P., Labarta A., Batlle X. (2010). Tuning the Size, the Shape, and the Magnetic Properties of Iron Oxide Nanoparticles. J. Phys. Chem. C.

[40] Raja K., Jaculine M.M., Jose M., Verma S., Prince A.A.M., Ilangovan K., Sethusankar K., Das S.J. (2015). Sol–gel Synthesis and Characterization of α-Fe_2_O_3_ Nanoparticles. Superlattices Microstruct..

[41] Kayani Z.N., Arshad S., Riaz S., Naseem S. (2014). Synthesis of Iron Oxide Nanoparticles by Sol–Gel Technique and Their Characterization. IEEE Trans. Magn..

[42] Jayanthi S.A., Nathan D.M.G.T., Jayashainy J., Sagayaraj P. (2015). A Novel Hydrothermal Approach for Synthesizing α-Fe_2_O_3_, γ-Fe_2_O_3_ and Fe_3_O_4_ Mesoporous Magnetic Nanoparticles. Mater. Chem. Phys..

[43] Ozel F., Kockar H., Karaagac O. (2014). Growth of Iron Oxide Nanoparticles by Hydrothermal Process: Effect of Reaction Parameters on the Nanoparticle Size. J. Supercond. Novel Magn..

[44] Zhang Q., Yang X., Guan J. (2019). Applications of Magnetic Nanomaterials in Heterogeneous Catalysis. ACS Appl. Nano Mater..

[45] Gama Cavalcante A.L., Dari D.N., Izaias da Silva Aires F., Carlos de Castro E., Moreira Dos Santos K., Sousa Dos Santos J.C. (2024). Advancements in Enzyme Immobilization on Magnetic Nanomaterials: Toward Sustainable Industrial Applications. RSC Adv..

[46] Wu W., He Q., Jiang C. (2008). Magnetic Iron Oxide Nanoparticles: Synthesis and Surface Functionalization Strategies. Nanoscale Res. Lett..

[47] Cui J., Tang X., Ma Q., Chang Y., Zhang Q., Jia S. (2024). Cross-Linked α-Amylase Aggregates on Fe_3_O_4_ Magnetic Nanoparticles Modified with Polydopamine/polyethyleneimine for Efficient Hydrolysis of Starch. Particuology.

[48] Feng Y., Hu H., Wang Z., Du Y., Zhong L., Zhang C., Jiang Y., Jia S., Cui J. (2021). Three-Dimensional Ordered Magnetic Macroporous Metal-Organic Frameworks for Enzyme Immobilization. J. Colloid Interface Sci..

[49] Bahri S., Homaei A., Mosaddegh E. (2022). Zinc Sulfide-Chitosan Hybrid Nanoparticles as a Robust Surface for Immobilization of Sillago Sihama α-Amylase. Colloids Surf. B Biointerfaces.

[50] Hojnik Podrepšek G., Knez Ž., Leitgeb M. (2020). Development of Chitosan Functionalized Magnetic Nanoparticles with Bioactive Compounds. Nanomaterials.

[51] Virgen-Ortíz J.J., dos Santos J.C.S., Berenguer-Murcia Á., Barbosa O., Rodrigues R.C., Fernandez-Lafuente R. (2017). Polyethylenimine: A Very Useful Ionic Polymer in the Design of Immobilized Enzyme Biocatalysts. J. Mater. Chem. B Mater. Biol. Med..

[52] Guisan J.M., Fernandez-Lorente G., Rocha-Martin J., Moreno-Gamero D. (2022). Enzyme Immobilization Strategies for the Design of Robust and Efficient Biocatalysts. Curr. Opin. Green Sustain. Chem..

[53] Yu C., Li Q., Tian J., Zhan H., Zheng X., Wang S., Sun X., Sun X. (2021). A Facile Preparation of Immobilized Naringinase on Polyethyleneimine-Modified Fe_3_O_4_ Magnetic Nanomaterials with High Activity. RSC Adv..

[54] Gough J.E., Scotchford C.A., Downes S. (2002). Cytotoxicity of Glutaraldehyde Crosslinked Collagen/poly(vinyl Alcohol) Films Is by the Mechanism of Apoptosis. J. Biomed. Mater. Res..

[55] Chalella Mazzocato M., Jacquier J.-C. (2024). Recent Advances and Perspectives on Food-Grade Immobilisation Systems for Enzymes. Foods.

[56] Singh I., Dhawan G., Gupta S., Kumar P. (2020). Recent Advances in a Polydopamine-Mediated Antimicrobial Adhesion System. Front. Microbiol..

[57] Hong S., Kim K.Y., Wook H.J., Park S.Y., Lee K.D., Lee D.Y., Lee H. (2011). Attenuation of the in Vivo Toxicity of Biomaterials by Polydopamine Surface Modification. Nanomedicine.

[58] Lee H., Dellatore S.M., Miller W.M., Messersmith P.B. (2007). Mussel-Inspired Surface Chemistry for Multifunctional Coatings. Science.

[59] Lee H., Rho J., Messersmith P.B. (2009). Facile Conjugation of Biomolecules onto Surfaces via Mussel Adhesive Protein Inspired Coatings. Adv. Mater..

[60] Ryu J.H., Messersmith P.B., Lee H. (2018). Polydopamine Surface Chemistry: A Decade of Discovery. ACS Appl. Mater. Interfaces.

[61] Luo R., Tang L., Wang J., Zhao Y., Tu Q., Weng Y., Shen R., Huang N. (2013). Improved Immobilization of Biomolecules to Quinone-Rich Polydopamine for Efficient Surface Functionalization. Colloids Surf. B Biointerfaces.

[62] Zheng X., Li Q., Tian J., Zhan H., Yu C., Wang S., Sun X. (2021). Novel Strategy of Mussel-Inspired Immobilization of Naringinase with High Activity Using a Polyethylenimine/Dopamine Co-Deposition Method. ACS Omega.

[63] Hoare D.G., Koshland D.E. (1967). A Method for the Quantitative Modification and Estimation of Carboxylic Acid Groups in Proteins. J. Biol. Chem..

[64] Fernandez-Lafuente R., Rosell C.M., Rodriguez V., Guisan J.M. (1995). Strategies for Enzyme Stabilization by Intramolecular Crosslinking with Bifunctional Reagents. Enzym. Microb. Technol..

[65] Lutterotti L., Scardi P. (1990). Simultaneous Structure and Size–strain Refinement by the Rietveld Method. J. Appl. Crystallogr..

[66] Lutterotti L., Matthies S., Wenk H. (1999). MAUD: A Friendly Java Program for Material Analysis Using Diffraction. Newsl. CPD.

[67] Scardi P., Lutterotti L., Maistrelli P. (1994). Experimental Determination of the Instrumental Broadening in the Bragg–Brentano Geometry. Powder Diffr..

[68] López-Gallego F., Montes T., Fuentes M., Alonso N., Grazu V., Betancor L., Guisán J.M., Fernández-Lafuente R. (2005). Improved Stabilization of Chemically Aminated Enzymes via Multipoint Covalent Attachment on Glyoxyl Supports. J. Biotechnol..

[69] Boudrant J., Woodley J.M., Fernández-Lafuente R. (2020). Parameters Necessary to Define an Immobilized Enzyme Preparation. Process Biochem..

[70] Sadana A. (1988). Enzyme Deactivation. Biotechnol. Adv..

[71] Delhez R., de Keijser T.H., Ian Langford J., Louër D., Mittemeijer E.J., Sonneveld E.J. (1993). The Rietveld Method.

[72] de Keijser T.H., Langford J.I., Mittemeijer E.J., Vogels A.B.P. (1982). Use of the Voigt Function in a Single-Line Method for the Analysis of X-Ray Diffraction Line Broadening. J. Appl. Crystallogr..

[73] Kim W., Suh C.-Y., Cho S.-W., Roh K.-M., Kwon H., Song K., Shon I.-J. (2012). A New Method for the Identification and Quantification of Magnetite–maghemite Mixture Using Conventional X-Ray Diffraction Technique. Talanta.

[74] Roth H.-C., Schwaminger S.P., Schindler M., Wagner F.E., Berensmeier S. (2015). Influencing Factors in the CO-Precipitation Process of Superparamagnetic Iron Oxide Nano Particles: A Model Based Study. J. Magn. Magn. Mater..

[75] Li Z., Chanéac C., Berger G., Delaunay S., Graff A., Lefèvre G. (2019). Mechanism and Kinetics of Magnetite Oxidation under Hydrothermal Conditions. RSC Adv..

[76] Ren Y., Rivera J.G., He L., Kulkarni H., Lee D.-K., Messersmith P.B. (2011). Facile, High Efficiency Immobilization of Lipase Enzyme on Magnetic Iron Oxide Nanoparticles via a Biomimetic Coating. BMC Biotechnol..

[77] Li G.-Y., Jiang Y.-R., Huang K.-L., Ding P., Chen J. (2008). Preparation and Properties of Magnetic Fe_3_O_4_–chitosan Nanoparticles. J. Alloys Compd..

[78] Zhu L., Lu Y., Wang Y., Zhang L., Wang W. (2012). Preparation and Characterization of Dopamine-Decorated Hydrophilic Carbon Black. Appl. Surf. Sci..

[79] Puri M. (2011). Updates on Naringinase: Structural and Biotechnological Aspects. Appl. Microbiol. Biotechnol..

[80] Bodakowska-Boczniewicz J., Garncarek Z. (2020). Immobilization of Naringinase from Aspergillus Niger on a Magnetic Polysaccharide Carrier. Molecules.

[81] Ribeiro M.H.L., Rabaça M. (2011). Cross-Linked Enzyme Aggregates of Naringinase: Novel Biocatalysts for Naringin Hydrolysis. Enzym. Res..

[82] Busto M.D., Meza V., Ortega N., Perez-Mateos M. (2007). Immobilization of Naringinase from Aspergillus Niger CECT 2088 in Poly(vinyl Alcohol) Cryogels for the Debittering of Juices. Food Chem..

[83] Norouzian D., Hosseinzadeh A., Inanlou D.N., Moazami N. (2000). Production and Partial Purification of Naringinase by Penicillium Decumbens PTCC 5248. World J. Microbiol. Biotechnol..

[84] Tsen H.-Y., Tsai S.-Y. (1988). Comparison of the Kinetics and Factors Affecting the Stabilities of Chitin-Immobilized Naringinases from Two Fungal Sources. J. Ferment. Technol..

[85] Zheng X., Li Q., Tian J., Zhan H., Yu C., Wang S., Sun X., Sun X. (2021). Facile Preparation of Immobilized Naringinase on Polyethylenimine/dopamine-coated Hydrothermal Carbon Spheres with High Performance. J. Chem. Technol. Biotechnol..

